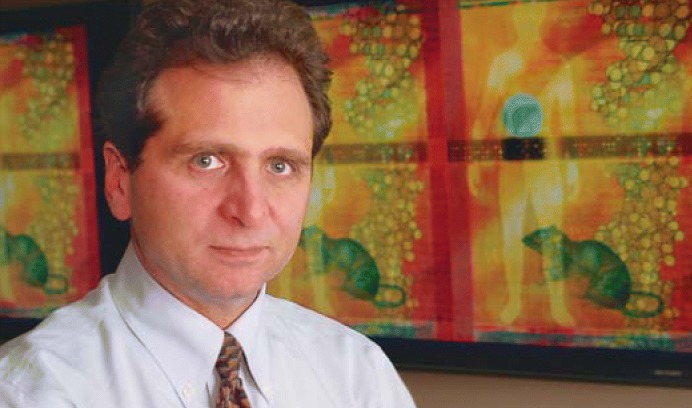# Environmental Genomics: An Opportunity for the NIEHS

**DOI:** 10.1289/ehp.114-a14

**Published:** 2006-01

**Authors:** David A. Schwartz

**Affiliations:** Director, NIEHS and NTP E-mail: david.schwartz@niehs.nih.gov

As I continue to consider new research opportunities for the NIEHS, my desire to support research in environmental genomics grows. While the accomplishments and available tools in genetics and genomics certainly enhance my enthusiasm for this field of research, my attraction to environmental genomics stems from my belief that environmental exposures can be used to understand the role of transcriptional regulation and genetic variation in the development and progression of common yet complex human diseases.

A growing body of research helps to illustrate the opportunities and challenges that lie before us. The influence of environmental exposures on transcriptional regulation of genes is clearly highlighted by the field of epigenetics. Michael Skinner at Washington State University and colleagues recently demonstrated the potential transgenerational adverse effects of intrauterine exposure to endocrine-disrupting pesticides on male fertility (Anway et al. 2005). Findings from Randy Jirtle’s laboratory at Duke University indicate that exposure through maternal diet to common methylating agents found in vegetables and vitamin supplements can have profound effects on gene expression in offspring that continue to be inherited in subsequent generations (Waterland and Jirtle 2003). Moreover, since monozygotic twins diverge in the concordance of methylation as a function of age (Fraga et al. 2005), it is abundantly clear that methylation is a dynamic process.

These findings underscore the role that intrauterine exposures could potentially have on common complex diseases that involve developmentally vulnerable organ systems. Such research also indicates that environmental exposures may serve as biological clues to understanding the regulation of gene expression and the role that transcriptional regulation may have on the risk of developing disease, as well as point to novel therapeutic interventions.

Environmental exposures can also be used to simplify complex biological processes to both discover unique biological mechanisms and narrow the pathophysiologic phenotype of complex human diseases. For instance, the discovery of the aryl hydrocarbon receptor (AhR) occurred as a direct result of the known toxicity of dioxin and polycyclic aromatic hydrocarbons. Not only did this discovery demonstrate the biological role of the AhR in mediating the toxicity to these agents, it also revealed the role of the AhR in homeostatic and basic pathophysiologic processes. Most importantly, however, the identification of the AhR led to the ultimate discovery of the PAS (PER-ARNT-SIMS) superfamily of receptors that mediate response to various forms of environmental stress such as hypoxemia and circadian rhythm, and control basic physiologic activities such as vascular development, learning, and neurogenesis (Kewley et al. 2004; Nebert et al. 2004).

Likewise, understanding of environmental exposures can simplify complex disease processes by narrowing the pathophysiologic phenotype to elucidate the genetics and biology that underlie a particular condition. For example, diseases such as asthma arise from dozens of etiologic agents. Since asthma caused or exacerbated by dust mites, endotoxin, or ozone involves different genes and different biological mechanisms, the disease can be better studied by focusing the investigation on a specific etiologic type of asthma.

Given that an extensive number of animal genomes have been sequenced and have demonstrated the evolutionary conservation of biology and genetic structure, comparative genomics will be an important tool for identifying the genes that control response to specific environmental agents, which in turn will accelerate our discoveries in environmental health sciences. For instance, the discovery of the importance of the toll-like receptors in innate immunity in mammals occurred as a direct result of the observation that a defective receptor in flies caused them to be much more susceptible to *Aspergillus fumigatus* (Lemaitre et al. 1996; Medzhitov et al. 1997). The ease with which we can observe and apply knowledge across model systems must be exploited so that we can efficiently understand the biological and clinical importance of environmentally responsive genes.

A clear challenge to the field of environmental health sciences will be to make the best use of environmental genomics to inform our understanding of the interaction between environmental exposures and genes in the development and progression of human diseases.

To facilitate progress in environmental genomics, we need to train young investigators in the discipline and support scientific programs that focus on biological and clinical problems that can most directly be solved by employing these novel conceptual and methodological approaches. However, to truly have an impact on human health, we need to extend these approaches to understanding chronic complex human diseases including cardiac disease, cancer, diabetes, chronic lung disease, and cerebrovascular disease. These diseases account for substantial morbidity and mortality worldwide, yet avoidable environmental exposures and reversible behaviors play a critical role in their development (Willett 2002). A clear challenge to the field of environmental health sciences will be to make the best use of environmental genomics to inform our understanding of the interaction between environmental exposures and genes in the development and progression of human diseases, and ultimately to translate this knowledge into effective prevention, intervention, and treatment strategies.

## Figures and Tables

**Figure:**